# How Instructions, Learning, and Expectations Shape Pain and Neurobiological Responses

**DOI:** 10.1146/annurev-neuro-101822-122427

**Published:** 2023-03-14

**Authors:** Lauren Y. Atlas

**Affiliations:** 1 National Center for Complementary and Integrative Health and National Institute of Mental Health, National Institutes of Health, Bethesda, Maryland, USA; 2 National Institute on Drug Abuse, National Institutes of Health, Baltimore, Maryland, USA

**Keywords:** pain, placebo, instructions, learning, expectations, neuroscience

## Abstract

Treatment outcomes are strongly influenced by expectations, as evidenced by the placebo effect. Meta-analyses of clinical trials reveal that placebo effects are strongest in pain, indicating that psychosocial factors directly influence pain. In this review, I focus on the neural and psychological mechanisms by which instructions, learning, and expectations shape subjective pain. I address new experimental designs that help researchers tease apart the impact of these distinct processes and evaluate the evidence regarding the neural mechanisms by which these cognitive factors shape subjective pain. Studies reveal that expectations modulate pain through parallel circuits that include both pain-specific and domain-general circuits such as those involved in affect and learning. I then review how expectations, learning, and verbal instructions impact clinical outcomes, including placebo analgesia and responses to pharmacological treatments, and discuss implications for future work.

## 1. INTRODUCTION

Expectations directly influence perception across numerous domains (de Lange et al. 2018), including clinical outcomes. The placebo effect describes the phenomenon whereby individuals experience beneficial health outcomes in response to inert treatments. Placebo effects are thought to depend on expectations attributed to the psychosocial treatment context, including prior experience and verbal instructions. Placebo effects are largest in pain [placebo analgesia (Hróbjartsson & Gøtzsche 2001, 2004)]. Placebo analgesia is often dismissed as response bias (Hróbjartsson et al. 2011) since pain is subjective and measured with self-report. However, neuroimaging and other tools allow researchers to probe neurobiological processes and isolate the mechanisms that construct subjective pain in different contexts. Understanding how pain is constructed is essential, as pain is the leading reason that individuals seek medical assistance (Schappert & Burt 2006), one out of five American adults suffers from chronic pain (Dahlhamer et al. 2018), and pain is central to the opioid epidemic (Volkow & McLellan 2016). If we determine how expectations and psychosocial factors shape pain, we might leverage these insights to ameliorate pain and perhaps even reduce reliance on pharmacological interventions.

In this article, I review brain mechanisms underlying expectancy-based pain modulation. I concentrate on two factors that give rise to expectations: learning and verbal instruction. I first address the neural mechanisms by which learning shapes expectations and pain. I then address the role of verbal instructions and how researchers can dissociate effects of instructions and learned expectations. I also discuss the role of explicit expectations and learning in the absence of awareness. Finally, I review how instructions, learning, and expectations contribute to placebo analgesia and clinical outcomes and address outstanding questions. I hope to motivate other neuroscientists to study pain and its modulation so we may reduce suffering and improve health.

## 2. LEARNING AND THE DEVELOPMENT OF EXPECTATIONS

Over a century of research has focused on classical conditioning and Pavlovian associative learning. While a thorough review of learning is beyond the scope of this article, pain and learning are inherently linked. Pain signals potential bodily damage, and therefore organisms are motivated to learn from pain to avoid harm and threats to their safety. The mechanisms associated with learning from aversive stimuli have been studied across species and are likely to be biologically prepared, as even low-level organisms such as zebrafish have cellular mechanisms that are specific to noxious stimulation and allow for avoidance of future harm (Ohnesorge et al. 2021). However, the extent to which simple organisms experience pain, rather than nociception, is widely debated. In this review, I focus on the subjective experience of pain measured in humans.

### 2.1. Learning to Predict Pain

**Figure 1:**
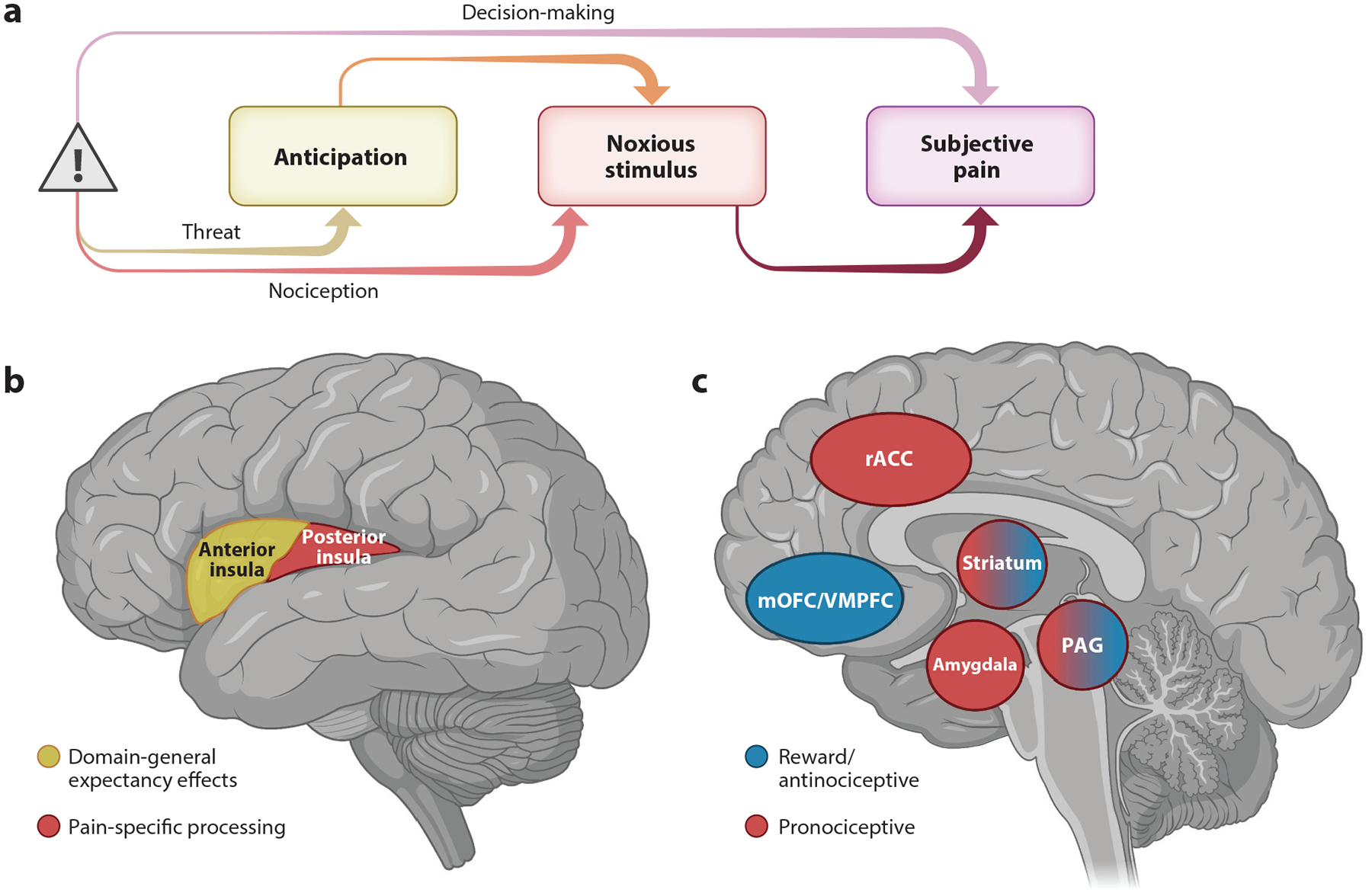
Brain mechanisms of expectancy-based pain modulation. (*a*) Neuroimaging studies of expectancy-based pain modulation pair predictive cues with noxious stimulation to determine how pain is influenced by threat, nociception, and decision-making. (*b*) Functional MRI studies that compare expectancy effects on pain with other aversive modalities (Fazeli & Büchel 2018, Horing & Büchel 2022, Sharvit et al. 2018) indicate that the anterior insula (*yellow*) is responsive to expectancies across domains, whereas posterior portions of the insula (*red*) seem to uniquely respond to pain. (*c*) Although dopaminergic circuits are most often studied in the context of reward and appetitive learning, these circuits are highly implicated in pain and aversive learning, as are opioidergic circuits. Mesolimbic circuits such as the medial prefrontal cortex, medial orbitofrontal/ventromedial prefrontal cortex (mOFC/VMPFC), and the ventral striatum, including the nucleus accumbens, respond to reward stimuli and generally show responses that are inversely related to pain, while the dorsal striatum shows both appetitive and aversive prediction errors (Seymour et al. 2005). Neurons in the periaqueductal gray (PAG) can facilitate or inhibit pain through opioidergic projections to the rostral ventral medulla, nucleus accumbens, and the amygdala (Fields 2006, 2018), and functional connectivity between the PAG and rostral anterior cingulate cortex (rACC) is linked to μ-opioid-based placebo analgesia (Bingel et al. 2006, Eippert et al. 2009a). Finally, the amygdala contains neurons involved in pain unpleasantness (Corder et al. 2019) and might respond uniquely to experiential threat learning (Atlas 2019), although the amygdala is also responsive to reward and reward learning (Murray 2007). All of these regions contain both μ-opioid and dopamine receptors. Panels *b* and *c* adapted from images created with Biorender.com.

**Figure 2:**
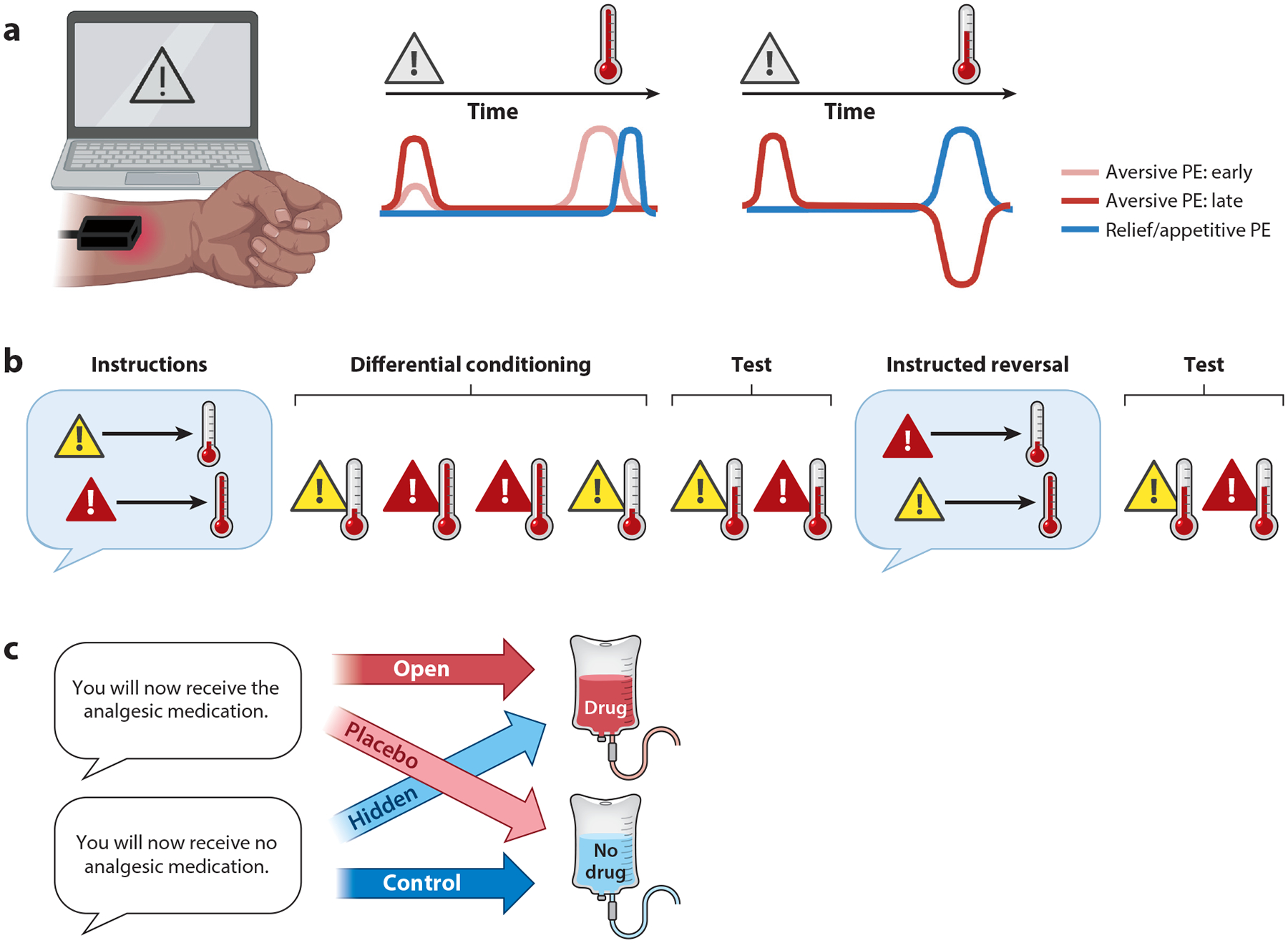
Experimental approaches to investigate the impact of learning and instructions on pain and analgesia. (*a*) Error-based pain learning. By pairing neutral cues with noxious stimulation, researchers can measure how expectations develop and vary over time. Here, a visual symbol warns about the delivery of a noxious thermal stimulus via a thermode on the participant’s forearm. Quantitative models of error-driven learning capture how expectations develop as a function of predictions and prediction errors (PEs). Early in learning (*pink*), heat is unexpected and thus elicits an aversive PE. An appetitive PE (*blue*) may occur at heat offset, consistent with relief. In late learning (*red*), cue-outcome contingencies have been reinforced, and the PE shifts to the time of the cue, representing the prediction itself. If a stimulus is delivered that is lower than expected (*right*), this may generate an appetitive PE and/or a reduction in the aversive PE. Panel *a* adapted from images created with Biorender.com. (*b*) Instructed reversal paradigm. By combining verbal instructions with conditioning and contingency reversals, researchers can tease apart their independent contributions to pain, autonomic responses, and brain activation. Here, individuals are instructed that the red cue predicts more pain than the yellow cue. These contingencies are reinforced during conditioning, and then during a test phase each cue is paired with stimulation of the same intensity to test for effects of instructions and learning. Following the test, individuals are instructed that contingencies have reversed, and then the cues are both paired with the same test stimulus. If responses reverse upon instruction, they depend on higher-order knowledge, whereas if they do not, they depend on experiential learning. (*c*) Balanced placebo design. The balanced placebo design crosses verbal instructions (*left*) with treatment delivery (*right*) in a 2 × 2 factorial design to test whether expectancy and treatment have additive or interactive effects on clinical outcomes such as pain.

The study of aversive learning in humans has flourished over the last century. While most fear conditioning studies employ stimuli such as electric shocks that are tailored to be aversive but not painful, I focus on how we learn about painful stimuli themselves. By presenting cues that predict painful outcomes (see Figure 1a) during functional magnetic resonance imaging (fMRI) scanning, neuroscientists can (*a*) isolate brain regions involved in pain anticipation, (*b*) determine how cue-based expectations about pain intensity shape brain responses to noxious stimulation, and (*c*) examine how threat, nociception, expectations, and decision-making drive subjective pain. In addition, by considering the precise dynamics of these relationships (Figure 2), researchers can determine how pain-related expectations develop and change across time (i.e., learning). While an in-depth discussion of individual studies is beyond the scope of this review, neuroimaging meta-analyses combine across studies to isolate core mechanisms involved in the anticipation of pain (Palermo et al. 2015) and aversive experiences (Andrzejewski et al. 2019) and to show how responses to noxious stimuli are modulated by expectations (Atlas & Wager 2014; Zunhammer et al. 2018, 2021).

Two main patterns emerge from these studies, which generally use fMRI to measure blood oxygen level–dependent (BOLD) responses to acute noxious stimulation. First, most brain regions that contain nociceptive neurons, that is, the so-called pain matrix (Derbyshire 2000), are modulated when cues induce expectations about the onset of pain or its intensity. During pain anticipation, elevated BOLD activation is observed in the bilateral insula, middle cingulate cortex, and medial thalamus, among other regions (Palermo et al. 2015). These same regions show reduced BOLD activation to noxious stimulation following expectations for low pain, relative to expectations for high pain or neutral expectations, whether through pain-predictive cues or placebo analgesia (Atlas & Wager 2014). Does this provide evidence that predictions modulate nociceptive processing? In fact, the regions that show the most reliable evidence for modulation by expectations—the insula, dorsal anterior cingulate cortex, and thalamus—are the most likely to be activated across any fMRI study (Yarkoni et al. 2011), are involved in the maintenance of task sets (Dosenbach et al. 2006), and are responsive to any salient stimuli (Uddin 2014) and thus collectively referred to as the salience network. Thus, expectations may shape pain by altering the salience of a noxious stimulus. To determine whether effects are specific to pain or reflect shifts in attention, arousal, and salience, tests must be sensitive and specific to pain (Lee et al. 2020). One promising approach is to use brain-based biomarkers such as the neurologic pain signature (NPS) (Wager et al. 2013), a pattern of weights that can predict whether or not activation reflects nociceptive pain. Pain anticipation does not affect the NPS (Wager et al. 2013), and anticipatory responses in emotion-related regions but not regions involved in pain or cognitive control predict the magnitude of placebo analgesia across individuals (Wager et al. 2011). Consistent with this, a meta-analysis of studies across multiple stimulus modalities (Andrzejewski et al. 2019) indicates that anticipation of aversive events recruits a network of regions, including the anterior insula, anterior and middle cingulate cortex, amygdala, caudate, thalamus, and dorsolateral prefrontal cortex (DLPFC), and that most of these regions respond similarly whether or not the event is tactile. However, some regions, including somatosensory cortex, thalamus, anterior insula, midbrain, and dorsomedial prefrontal cortex, were preferentially active during anticipation of tactile events, whereas other regions such as the amygdala and hippocampus were more evident during the anticipation of aversive visual stimuli (Andrzejewski et al. 2019). To further understand whether these responses are unique to pain, we must relate responses during anticipation with pain itself and compare responses across modalities, as discussed in Section 2.3.

Computational models of associative learning provide further insight on systems involved in pain anticipation. Models of error-driven learning describe how we develop associations between predictive cues and painful outcomes and how predictions update dynamically in response to unexpected outcomes (i.e., prediction errors; see Figure 2a). Computational neuroscience studies reveal that many circuits implicated in reward learning play similar roles when organisms learn about pain (see Figure 1c). In a series of influential studies, Seymour et al. (2004, 2005) demonstrated that the lateral orbitofrontal cortex (OFC), putamen, insula, ventral striatum, and brainstem are sensitive to error-driven learning when cues are probabilistically related to pain or pain relief. Moreover, direct comparisons between pain exacerbation and pain relief during tonic pain (Seymour et al. 2005) revealed that aversive prediction errors (worse-than-expected outcomes) were associated with activation in the bilateral lateral OFC and the subgenual ACC, appetitive prediction errors (better-than-expected outcomes) were associated with activation in the amygdala and left substantia nigra, and the putamen, anterior insula, and rostral anterior cingulate cortex (rACC) were sensitive to both types of prediction errors. Other studies have shown that learning about pain can vary dynamically as a function of associability, which varies as a function of attention and uncertainty (Pearce & Hall 1980), and that when models are fit to anticipatory skin conductance responses (a measure of arousal), the striatum tracks prediction error while the amygdala tracks associability (Zhang et al. 2016). For a thorough discussion on quantitative learning models of pain, see the review by Seymour (2019). The presence of appetitive prediction errors in response to pain relief supports the proposal that there may be a common currency of value (Dickinson & Dearing 1980, Levy & Glimcher 2012) and that pain can be compared with primary and secondary reinforcers such as juice rewards and money (Becker et al. 2017, Park et al. 2011, Talmi et al. 2009). Interactions between pain and reward are complex and take different forms; these relationships have been discussed at length in other reviews (Borsook et al. 2016, Leknes & Tracey 2008, Navratilova et al. 2015).

### 2.2. Learning to Avoid Pain

What happens when we predict that pain will occur? A natural goal is to avoid potential harm. Whereas Pavlovian studies measure passive learning, pain avoidance can be studied using operant conditioning and instrumental learning. For example, participants might choose between cues that are probabilistically linked to the delivery or omission of painful thermal stimulation. Roy et al. (2014) found that aversive prediction errors were associated with activation of the periaqueductal gray (PAG), a key brainstem structure involved in opioid release and descending pain modulation (Fields 2004) that can flexibly regulate approach and avoidance behaviors (Reis et al. 2021) and facilitate escape (Lefler et al. 2020). This suggests that the PAG contributes not only to automatic, preprogrammed behaviors (which are likely to be influenced by Pavlovian learning) but also to instrumental behaviors. A recent study that compared learning from experienced pain and avoided pain (Jepma et al. 2022) replicated these findings and also observed associations between aversive prediction errors and rACC activation. PAG-rACC connectivity (Bingel et al. 2006, Eippert et al. 2009a) is thought to underlie endogenous opioid-based pain modulation during placebo analgesia (see the sidebar titled The Role of Endogenous Opioids). The rACC was also implicated in a study of active relief learning (Zhang et al. 2018) in which individuals learned to temporarily relieve (and thus avoid) tonic pain. The rACC tracked associability, while the dorsal putamen tracked prediction error. Although Jepma et al. (2022) did not observe striatal prediction errors, these may vary across individuals during avoidance learning depending on whether individuals learn more from pain avoidance or from pain experience (Eldar et al. 2016).

The networks involved in pain avoidance learning, including the PAG, rACC, and striatum (Figure 1c), are implicated in endogenous opioid and dopamine signaling across species (Bromberg-Martin et al. 2010, Nummenmaa & Tuominen 2018). Jepma et al. (2022) tested whether these neuromodulators impact pain avoidance by administering the opioid antagonist naltrexone or the dopamine agonist levodopa during learning. Individuals in a control group learned more quickly from received pain than from outcomes they avoided, whereas both drugs reduced this discrepancy by increasing learning rates specifically for avoided stimuli (although neither drug impacted brain responses). Desch et al. (2022) also recently found that both levodopa and naltrexone enhance relief learning relative to placebo. It might seem surprising that both drugs elicit effects in the same direction since levodopa increases dopamine levels whereas naltrexone reduces opioid binding. However, μ-opioid agonists can have both excitatory and inhibitory effects on dopamine neurons (Margolis et al. 2014), and thus an opioid antagonist might have the same impact as a dopamine agonist. As discussed in Section 5, more work is needed on how endogenous opioids and dopamine contribute to pain avoidance, relief, and pain modulation. In addition, future studies should explore how dopamine and opioids contribute to Pavlovian learning using similar approaches.

### 2.3. How Learning Impacts Subjective Pain

Studies of Pavlovian and instrumental learning focus primarily on responses to cues that predict pain. How does the learning process shape pain itself? Insight can be achieved by pairing cues that predict different intensities with a single noxious stimulus (see Figure 2b) and comparing pain and brain responses to the same stimulus as a function of cue. Numerous studies indicate that pain reports are biased toward predictive cues (Atlas et al. 2010, Brown et al. 2008a, Wiech et al. 2014) and that cues can modulate brain activation in response to noxious stimulation in most pain-related regions (Atlas et al. 2010; Brown et al. 2008a,b; Keltner 2006; Koyama et al. 2005; Ploghaus et al. 2001; Wiech et al. 2010). Atlas et al. (2010) used multilevel mediation (MacKinnon et al. 2007) to understand how these effects were linked, that is, which brain regions formally mediate cue effects on subjective pain. Participants were instructed about cue-heat contingencies and then underwent conditioning in which thermal stimuli calibrated to elicit low pain were paired with low-pain cues and high-intensity stimuli were paired with high-pain cues. Following conditioning, cues were paired with their predicted heat intensity or with a single temperature calibrated to elicit medium pain. Pain was reduced by 20% when medium heat was paired with low cues relative to high cues, and every region that tracked changes in temperature also exhibited reduced activation to medium heat with low cues relative to high cues. Responses in temperature-sensitive portions of the insula, dorsal anterior cingulate cortex, and thalamus formally mediated the trial-by-trial relationship between predictive cue and subjective pain in medium-heat trials. Cue effects on these mediator regions were in turn mediated by cue-evoked anticipatory responses in the striatum and ventromedial prefrontal cortex (VMPFC), regions involved in value-based learning across domains. These findings further link cue effects on pain with the learning mechanisms reviewed above. But are these mechanisms unique to pain?

We can test whether modulatory mechanisms are unique to pain by (*a*) using brain-based tests that are sensitive and specific to pain [e.g., the NPS (Wager et al. 2013)] and (*b*) comparing painful stimuli with other modalities. Interestingly, while predictive cues can alter NPS expression (Jepma et al. 2018, Woo et al. 2017), indicating that cue-based expectations can modulate pain-specific brain responses, placebo administration reduces pain but does not impact the NPS, suggesting nonnociceptive mechanisms (Zunhammer et al. 2018). This indicates that expectancy effects on pain may be mediated by different mechanisms depending on the type of expectation. I return to this topic in Section 5. Importantly, pain is a subjective experience, and while brain-based tests such as the NPS provide objective, quantifiable measures that are correlated with pain, they cannot replace pain ratings per se; they simply tell us how pain might be mediated. Comparing pain with other modalities provides additional insight. Three studies that compared cues that predict pain with cues that predict other unpleasant outcomes, such as odors (Sharvit et al. 2015, 2018), images (Fazeli & Büchel 2018), or sounds (Horing & Büchel 2022), all came to the same conclusion: A portion of the anterior insula is modulated by cues regardless of modality, whereas the posterior insula is uniquely sensitive to cues that predict pain (see Figure 1b). This is consistent with studies suggesting that the dorsal posterior insula might be specific to pain (Segerdahl et al. 2015) and that there are anterior-posterior gradients along the insula that subserve general interoception and thermosensation, respectively (Craig 2009). Thus, cue-based expectations seem to modulate both pain-specific and domain-general brain responses.

While most studies of predictive cue effects on pain maintain relatively stable cue-outcome contingencies, any subjective decision can vary in confidence, and confidence might moderate the extent to which we trust expectations. Indeed, pain is biased more strongly by cues that predict a certain outcome than by cues that signal uncertainty (Brown et al. 2008a), and confidence in one’s belief is associated with anticipatory responses in the right anterior insula, which in turn predicts the extent to which cues modulate subjective pain (Brown et al. 2008b). The predictive coding framework (Friston & Kiebel 2009) provides a theoretical and computational account for how a prediction’s uncertainty—or precision—impacts perception. Predictive coding suggests that the brain’s goal is to reduce the discrepancy between predictions and outcomes in the world, that is, prediction errors. With precise predictions about outcomes, our prediction strongly guides our responses, and we learn from the outcome whether our prediction was correct; otherwise we update based on prediction errors. When a prediction is less certain, we integrate the prediction with the outcome and rely less on our beliefs. Bayes’ rule provides a computational account for the integration of the prior (i.e., the prediction) with the likelihood of the outcome depending on the precision (i.e., uncertainty) of both estimates (Friston et al. 2014). Given that pain is subjective and impacted by predictions and uncertainty, researchers have proposed that predictive coding can account for placebo analgesia and expectancy effects on pain (Büchel et al. 2014, Ongaro & Kaptchuk 2019). Grahl et al. (2018) formally tested how precision impacts pain and placebo analgesia. Precision was positively associated with placebo analgesia (i.e., larger pain reduction with higher precision) but inversely related to activation in the PAG and the rostroventral medulla, another key node in the endogenous opioid system (Fields 2004). This result is consistent with other work that finds associations between experimentally manipulated uncertainty and PAG activation (Yoshida et al. 2013) but conflicts with observations of positive associations between PAG activation and placebo-related reductions (Atlas & Wager 2014). Nonetheless, this finding indicates that precision and uncertainty can influence whether expectations shape pain and pain-related brain responses.

fMRI provides important insights on associations between expectations, learning, pain, and brain responses. However, fMRI analyses are correlational and cannot reveal whether a brain region is necessary for pain modulation. This must be accomplished through tools that perturb brain activity and measure behavior. One key region is the VMPFC, which is implicated in flexible learning and value-based decision-making across affective modalities (Murray et al. 2007, Roy et al. 2012, Schneider & Koenigs 2017, Schoenbaum et al. 2007). We asked whether the VMPFC contributes causally to pain and its modulation by expectation by comparing individuals with bilateral VMPFC lesions following surgical resection to a group of healthy controls with intact brains (Motzkin et al. 2021). All participants underwent quantitative sensory testing to evaluate pain sensitivity and then completed a cue-based expectancy task based on the paradigm described above (Atlas et al. 2010), in which individuals were instructed about cue contingencies, contingencies were reinforced through conditioning, and then during a test phase, cues that predicted high or low heat were intermittently paired with heat calibrated to elicit moderate pain. Groups did not differ in pain sensitivity, pain unpleasantness, or the correspondence between pain and autonomic activity, suggesting that impairments to VMPFC function do not affect pain or nociception. However, relative to the control group, individuals with VMPFC lesions reported stronger cue effects on expectations both immediately after instructions and throughout the test phase. Individuals with VMPFC lesions also reported larger effects of cues on subjective pain in response to medium heat during the test phase, particularly for ratings of pain unpleasantness (Motzkin et al. 2021). This provides evidence that the VMPFC is not necessary for the development of expectations since expectations updated based on verbal instruction and modulated subjective pain. We hypothesize that group differences reflect the VMPFC group’s failure to update expectations when unexpected medium heat was delivered during the test phase (which requires error-based learning), and thus pain reports continued to reflect the rules that were instantiated through initial instructions. This is consistent with the VMPFC’s critical role in extinction learning (Gottfried & Dolan 2004, Phelps et al. 2004, Quirk et al. 2000) and learning latent rules (Takahashi et al. 2013, Wilson et al. 2014). However, because instructions and learning were combined, we cannot tease apart their potentially separate contributions, that is, whether individuals with VMPFC lesions showed stronger effects of instructions or less new learning when medium heat was introduced. To identify the unique impact of learning itself, we need to dissociate learning from verbal instruction and conscious expectations.

## 3. ISOLATING THE IMPACT OF INSTRUCTION, EXPECTATION, AND EXPERIENCE

While expectations can be learned through experience, they can also be influenced prior to experience in humans through verbal instruction. In addition, even when individuals are not instructed about contingencies, they are likely to develop explicit expectations about cue-outcome pairings. Therefore many effects reviewed above may reveal how conscious knowledge and explicit expectations shape pain rather than effects of learning per se. Historical debates focused on whether placebo effects were driven by expectancies or learning, but we now start from the broadly accepted assumption that learning contributes to expectations (Kirsch 1985). Careful experimental design allows researchers to differentiate between expectations that are learned through experience and those that develop through verbal instruction. In this section I focus on studies of cue-based pain modulation that evaluate whether dissociable mechanisms support pain modulation through instruction or experience.

### 3.1. Instructed Reversals Dissociate Learning and Verbal Instruction

Contingency reversals provide a powerful way to dissociate the impact of learning and verbal instruction. As introduced by Grings et al. (1973), individuals undergo standard fear conditioning and are then told that contingencies have reversed (Figure 2b). If conditioned responses reverse upon instruction, they are sensitive to learning and instructed knowledge; if they reflect original contingencies, they are sensitive to learning alone. Studies of aversive learning indicate that defensive responses (e.g., skin conductance, heart rate, pupil dilation) can reverse immediately upon instruction (Atlas & Phelps 2018, Costa et al. 2015, Grings et al. 1973). We measured whether instructed reversals impact brain responses during aversive learning (Atlas et al. 2016). Two groups of participants underwent the same reversal-learning task in which cues were paired with unpleasant but nonpainful shocks in 30% of trials. One group was informed about contingencies and reversals, while the other learned purely through experience. This allowed us to measure how instructions shape error-based learning, since both groups experienced the same cue-outcome pairings. Using a novel computational model that measures whether expectations that develop through error-based learning update upon instruction, we found that systems involved in value-based learning, including the striatum and VMPFC, were sensitive to instructions, consistent with findings in instructed reward learning (Doll et al. 2009, Li et al. 2011). Salience networks also updated immediately upon instruction. However, the amygdala, a critical region for threat acquisition and expression, tracked learning regardless of instruction, with similar dynamics whether individuals were instructed or learned purely through experience (Atlas et al. 2016, 2019). Thus some circuits are sensitive to higher-order knowledge, whereas others may be sensitive purely to experience. Multivoxel pattern analyses revealed similar findings in the amygdala (Braem et al. 2017). For more on instructed learning and the potentially unique role of the amygdala, see the review by Atlas (2019).

We recently used the same approach to measure how these processes shape subjective pain (Atlas et al. 2021). An uninstructed group of participants underwent pairings between pain-predictive cues and heat, and cue-outcome contingencies reversed three times. A second group experienced the same pairings but was informed about contingencies and reversals. Pain, autonomic responses, and responses to noxious stimuli in pain-related regions reversed as contingencies changed, and frontal regions, including the VMPFC and DLPFC, mediated cue effects on subjective pain in both groups. However, our instructed learning models (Atlas et al. 2016) revealed that learning-related responses in the striatum, VMPFC, and rACC differed depending on whether individuals were instructed about contingencies, such that these regions showed positive correlations with expected value in the instructed group, and negative correlations in the uninstructed group. Thus, even if pain is flexible and sensitive to both instructions and experience, instructions may alter the mechanisms by which individuals learn from experience. I consider how these factors in turn impact placebo responses in Section 4.

### 3.2. Self-Reported Expectations Shape Learning and Pain

As mentioned above, instructions and learning both contribute to the development of expectations, which in turn shape pain. But expectations are complex cognitions that may not strictly adhere to our experimental manipulations. For instance, the Gambler’s fallacy describes a phenomenon whereby individuals expect to lose after a series of wins or win after a series of losses (Ayton & Fischer 2004). These beliefs contradict what would be expected based on pure association and classic models of error-driven learning. Can we capture the impact of expectations themselves, particularly if they may deviate from experimental manipulations?

Measuring expectancy ratings provides insight on whether self-generated expectations influence aversive learning and subjective pain. Jepma et al. (2018) showed that computational learning models can account for how pain-predictive cues and pain outcomes influence subjective expectations and how these in turn update subjective pain. Pain was influenced by expectations above and beyond the effect of cues, consistent with a confirmation bias, and individual differences in confirmation bias moderated the association between prediction error and anticipatory activation in the striatum, VMPFC, PAG, insula, and other regions. This indicates that self-generated expectations can shape learning-related responses beyond pure associative mechanisms (Jepma et al. 2018).

What if reflecting on expectations changes learning? Although placebo researchers encourage clinicians and clinical trialists to ask patients about their expectations (Frisaldi et al. 2017), focusing on expectations might alter how we monitor our health. Comparing expectancy ratings with a no-rating condition indicates that ratings can indeed modulate learning. Individuals with amygdala lesions do not show differential skin conductance responses (SCRs) during pure Pavlovian aversive learning (Anderson & Phelps 2001) but do display differential SCRs when they provide expectancy ratings (Coppens et al. 2009), indicating that ratings engage circuits that bypass the amygdala. We compared participants who provided expectancy ratings during aversive reversal learning with a group who learned passively (Atlas et al. 2022). The rating group was slower to reverse SCRs when contingencies changed and expectancy ratings fully mediated cue effects on SCR. These studies suggest that rating expectations engages higher-order beliefs that can supersede pure associations and alters learning mechanisms. Future work should directly compare self-generated expectations with instruction-based knowledge.

### 3.3. Learning and Pain Modulation in the Absence of Explicit Knowledge

The studies discussed above allow individuals to generate explicit expectations about cue-outcome contingencies through learning or instruction. What if learning happens outside of explicit awareness? Some studies of associative learning suggest that learning can occur in the absence of explicit knowledge and that dissociable brain circuits are sensitive to associative learning versus higher-order knowledge (Clark et al. 2001, 2002; Mineka & Ohman 2002), with the amygdala being necessary for the expression of conditioned responses and the hippocampus being necessary for contingency awareness (Bechara et al. 1995). However, this dual systems model is widely debated (Lovibond & Shanks 2002, Mitchell et al. 2009), and recent meta-analyses indicate no evidence for unaware fear conditioning (Mertens & Engelhard 2020).

Nonetheless, pain is distinct from threat, and thus we can still evaluate whether pain-related learning can occur without explicit expectations or awareness. Jensen et al. (2012, 2015a,b) tested whether implicit cues (i.e., those presented briefly enough to be considered subliminal) shape pain in a series of papers. In the first study (Jensen et al. 2012), heat was paired with overt emotional faces during conditioning. During the test phase, faces were presented for 12 ms and masked by a scrambled image to preclude recognition. Relative to a control cue, pain was higher following subliminal presentations of the cue that was conditioned with high heat, and pain was lower following the cue that was conditioned with low heat, even though participants could not distinguish cues in post-task assessments. A follow-up study showed that subliminal cue effects on the right amygdala were correlated with effects on pain (Jensen et al. 2015a). While these studies presented overt images during conditioning, later studies evaluated subliminal cues during conditioning. Jensen et al. (2015b) found no impact of cue type on analgesia or hyperalgesia; however, Liu et al. (2020) measured responses in a larger sample and found that analgesic effects were only obtained with overt conditioning, whereas both subliminal and supraliminal conditioning led to hyperalgesia. Thus, separate mechanisms may support analgesia and hyperalgesia, and consistent with Jensen’s findings, sensitivity to subliminal cues that predict aversive outcomes may depend on the right amygdala. The right amygdala’s role in subliminal cue effects might link nonconscious pain modulation with work on the right amygdala’s unique sensitivity to experiential learning (Atlas et al. 2016, Braem et al. 2017).

## 4. HOW INSTRUCTIONS, LEARNING, AND EXPECTATIONS SHAPE PAIN IN THE CLINIC AND DURING PLACEBO ANALGESIA

Pain-predictive cues help researchers evaluate how instructions, learning, and expectations dynamically shape pain, behavior, and neurobiological responses. Cues acquire significance through learning or instruction and represent predictive features in the context surrounding pain. Of course, one of the most pertinent contexts for pain is the clinical context. Thus the impact of expectations is perhaps most significant in terms of how expectations shape pain during clinical encounters. This is most evident during placebo analgesia, in which pain is reduced due to the psychosocial context surrounding treatment, including patient expectations, emotions, and the patient-provider interaction. Studies of placebo analgesia administer placebos in the form of topical ointments, pills, and other inert interventions and measure whether pain and pain-related responses are modulated relative to control conditions. Many review papers have covered the general psychological and neurobiological mechanisms of placebo analgesia (Atlas 2021, Büchel et al. 2014, Colloca & Benedetti 2005, Geuter et al. 2017, Wager & Atlas 2015). In this section, I focus on how instructions and learning contribute to placebo analgesia and clinical outcomes.

### 4.1. The Impact of Instructions and Learning on Placebo Analgesia and Nocebo Hyperalgesia

Several studies have measured how instructions and learning contribute to placebo analgesia and its converse, nocebo hyperalgesia (i.e., pain enhancement based on expectations). Individuals who are instructed about a putative treatment and receive conditioning (i.e., reduction of a noxious stimulus on a placebo-treated site) show stronger placebo analgesia than those who receive instructions or conditioning alone (Colloca et al. 2008b), whereas nocebo hyperalgesia is similar regardless of whether individuals receive instructions or conditioning (Colloca et al. 2008a). These dissociations may point to differences in the mechanisms involved in pain relief versus pain exacerbation, consistent with other studies (Liu et al. 2020, Seymour et al. 2005).

Placebo studies have also leveraged instructed reversals to tease apart the contributions of instructions and learning. In one study (Benedetti et al. 2003), participants were conditioned with active treatments (ketorolac tromethamine to reduce pain, subthalamic nucleus stimulation in Parkinson’s disease, or sumatriptan, which affects cortisol and growth hormone). After several days, half of each group was told they would receive a new treatment with opposite effects (e.g., half the ketorolac group was told the new treatment would increase pain). Critically, all participants received placebo. Instructions reversed placebo effects on pain and motor performance but not on cortisol or growth hormone. This finding suggests that some outcomes—particularly conscious outcomes that can be monitored—may be more sensitive to instruction than others. Interestingly, the duration of conditioning might impact whether pain reverses upon instruction, as instructions that a treatment was inert reduced placebo effects in individuals who underwent one day of placebo conditioning but not in those who were conditioned for a full week, even though expectations did not differ between groups (Schafer et al. 2015). Thus, experience and instructions might interact to shape placebo analgesia.

While behavioral studies have separately examined instructions and conditioning, most fMRI studies combine both to maximize expectations prior to scanning. However, some studies rely on instructions alone. Schmid et al. (2013) instructed two groups that they would receive saline or an active treatment. The placebo group was told that they would receive a spasmolytic drug that would reduce pain, and the nocebo group was told they would receive naloxone, which would enhance pain. Both groups received visceral stimulation through a rectal balloon during fMRI scanning, and treatment was not administered. The groups reported similar pain during the saline control. During treatment, the nocebo group reported higher pain and worse expectations than the placebo group, and expectations were correlated with reported pain. Brain responses to visceral stimulation also differed between groups. Relative to the saline condition, nocebo instructions increased bilateral insula activation, whereas placebo instructions reduced bilateral insula activation. Direct comparisons revealed that somatosensory cortex activation was higher in the nocebo group than in the placebo group. A follow-up study (Schmid et al. 2015) observed similar instructed nocebo effects on the insula, as well as on the amygdala and thalamus. These studies provide important preliminary evidence that instructions can induce placebo and nocebo effects and modulate responses to noxious stimuli in pain-related regions. Future studies should leverage larger samples and different pain models to systematically isolate the contribution of instructions and learning to placebo analgesia and nocebo hyperalgesia.

### 4.2. Instructions Shape Responses to Active Treatments

Placebo analgesia reflects pain reduction with inert agents. We can also measure how expectations shape responses to active treatments, including analgesic drugs, and test assumptions that underlie placebo-controlled clinical trials. Randomized controlled trials (RCTs) compare a treatment arm with a placebo arm and assume that expectations and other nonspecific factors (e.g., natural history, regression to the mean, the psychosocial context) are identical across groups and additive with treatment effects. Differences between groups are thus attributed to the treatment. However, if treatments work differently when individuals believe they are receiving them, or if expectations affect outcomes differently when individuals receive treatment, then subtraction would over- or underestimate drug effects. By manipulating instructions alongside drug delivery, researchers can examine how expectations combine with actual drugs and test for interactions.

In the balanced placebo design (Rohsenow & Marlatt 1981), instructions about drug delivery are crossed with actual drug administration in a standard 2 × 2 factorial design (Figure 2c). Thus, participants receive treatment when they know they are receiving it (open administration) or when they believe they are receiving an inert agent (hidden administration), and participants receive an inert agent when they know they are receiving it (control) or when they believe they are receiving treatment (placebo). The design was initially developed to study alcohol (Rohsenow & Marlatt 1981) and has been applied to other treatments, including analgesics (Atlas et al. 2012, 2013; Butcher & Carmody 2012; De Vita et al. 2022; Schenk et al. 2014), nicotine (Gu et al. 2015, Juliano et al. 2011), and other substances (Juliano et al. 2019). We used a balanced placebo design to study remifentanil, a fast-acting opioid analgesic, and found that drug and expectancy effects on pain were additive (Atlas et al. 2012, 2013). These results support the assumption of additivity that underlies the RCT. Importantly, different treatments and different outcomes may be differentially sensitive to instruction-based expectations. In one study (Schenk et al. 2014), expectations enhanced pain when participants received lidocaine but not when they received placebo, and BOLD responses to noxious stimuli in the anterior insula, subgenual cingulate, and striatum showed similar interactions. We also observed interactions when we measured remifentanil effects on attention (Atlas et al. 2013) and observed pure drug effects regardless of instruction when we measured emotion perception. Balanced placebo designs should be used to test other analgesics and to determine how factors like drug dosage affect additivity. Studying the active treatment arm alone and comparing overt and covert drug administration can also provide insights (Bingel et al. 2011, Colloca et al. 2004), particularly when combined with pharmacological fMRI, which leverages pharmacokinetics and pharmacodynamics to isolate drug-induced brain changes (Atlas et al. 2012, Wise 2002). Comparing overt and covert administration provides an ethical advantage to studying expectancy in patient groups, as patients do not forego treatment. Notably, both the balanced placebo design and comparisons between overt and covert drug administration focus purely on the impact of instructions, regardless of learning history or prior experience. An important question is how treatment history impacts drug responses and instruction-based expectations.

## 5. OUTSTANDING QUESTIONS

Pain neuroscientists have leveraged modern technology and new computational advances to gain insights on pain and its modulation. Despite tremendous progress, many questions remain unanswered. In this section, I identify relevant areas of active research and outstanding questions.

### 5.1. Effects of Treatment History and Lived Experience

This review focuses on mechanistic studies of acute pain in healthy volunteers, which provides insight on pain that nearly everyone experiences. However, we must also understand how expectations affect chronic pain. Individuals with chronic pain have extended experience with pain and thus likely have different expectations from pain-free volunteers. Meta-analysis indicates that verbal instructions have moderate effects on acute pain in individuals with chronic pain (Peerdeman et al. 2016). Direct comparisons indicate that individuals with chronic musculoskeletal pain show similar placebo effects on acute pain and chronic pain, although the two are not correlated (Müller et al. 2016), and that the magnitude and reliability of placebo analgesia are equivalent between healthy controls and individuals with fibromyalgia and osteoarthritis (Power et al. 2020). However, other studies suggest that patients show reduced differential fear conditioning relative to controls (Harvie et al. 2017), and fear is linked to avoidance in chronic pain (Vlaeyen et al. 2016, Zaman et al. 2015). Chronic pain is also heterogeneous, comorbid with disorders of affect and substance use, and subject to health disparities. Thus, patients with different etiologies and treatment histories may have unique expectations about pain and treatment. Interestingly, chronic pain patients with more negative treatment histories showed slightly larger placebo effects on chronic pain (Müller et al. 2016), perhaps due to greater desire for relief, which is also linked to placebo (Vase et al. 2005). Addressing patients’ goals and expectations through an individualized, person-centered approach might improve patient outcomes. Placebo researchers have put together guidelines for health-care providers based on the current science (Evers et al. 2018, 2021), but more research is needed to determine which patients will respond to which types of expectations.

### 5.2. Patient-Provider Interactions and Socially Mediated Expectations

While a clinician’s verbal instructions can influence expectations, features of the patient-provider relationship contribute to clinical outcomes beyond the doctor’s words alone. New work is beginning to identify how social factors shape pain. Substantial psychological and neural overlap between physical pain and social distress has led to a great deal of interest in the concept of social pain, which has been addressed in previous reviews (Eisenberger 2015, Iannetti et al. 2013, Mogil 2015). In addition, recent studies indicate that expectations may vary depending on features of the provider (Howe et al. 2017, Necka et al. 2021) and that the patient-provider interaction can impact pain relief (Anderson et al. 2020, Kaptchuk et al. 2008, Reynolds Losin et al. 2017). We should continue to pursue work on not only the mechanisms by which instructions shape pain, placebo, and responses to treatment but also how the person delivering instructions impacts responses. For an excellent discussion of socially mediated expectations and their contribution to pain, see the review by Koban et al. (2017).

### 5.3. Are Mechanisms Specific to Pain?

As mentioned above, researchers have begun to evaluate specificity in terms of both whether placebos shape pain-specific responses (Zunhammer et al. 2018) and how expectancy effects on pain compare to other modalities (Fazeli & Büchel 2018, Horing & Büchel 2022, Sharvit et al. 2018). These studies suggest that many modulatory mechanisms are unlikely to be specific to pain. These findings probably reflect general principles by which expectations shape perception, including value-based learning, emotion regulation, and decision-making, as discussed in other reviews (Ashar et al. 2017, Atlas 2021, Wager & Atlas 2015, Wiech 2016). But certain mechanisms are likely unique, including posterior insula responses (Horing & Büchel 2022, Segerdahl et al. 2015), descending opioid engagement (Eippert et al. 2009a, Levine et al. 1978, Scott et al. 2008, Wager et al. 2007, Zubieta 2005), and peripheral responses such as spinal reflexes (Goffaux et al. 2007). I focused primarily on fMRI studies in humans in the present review, but multimodal investigations provide insights on the extent to which cortical responses to instructions, learning, and expectations can impact peripheral responses that may be unique to pain or depend on different neuromodulators such as opioids and dopamine. Finally, preclinical and nonhuman primate models provide insights on the circuit mechanisms that underlie pain-related learning (Johansen & Fields 2004, Lee et al. 2015), avoidance (Machado et al. 2009, Reis et al. 2021) and relationships between pain and reward (Navratilova et al. 2012). However, only human studies can reveal the impact of verbal instructions.

## 6. CONCLUSION

Instructions and learning influence pain and responses to treatment. Through careful experimental designs, neuroscience reveals how knowledge can interact with experience to shape pain, brain responses, and clinical outcomes. This may provide a window on how clinicians can leverage the psychosocial treatment context to improve patient outcomes. To achieve this goal, future studies should determine which outcomes, which patients, and which treatments are most sensitive to contextual and psychological factors such as expectations.

## THE ROLE OF ENDOGENOUS OPIOIDS

Endogenous opioids have been implicated in placebo analgesia since the discovery that placebo analgesia was reduced in dental patients who received the μ-opioid antagonist naloxone (Levine et al. 1978). These findings have been replicated (Benedetti 1996) and extended through multiple techniques. Positron emission tomography studies use [11^c^]carfentanil, a μ-opioid-selective radioligand, to identify where placebos enhance opioid binding (Wager et al. 2007, Zubieta 2005). Others show that opioid analgesics and placebo have common effects on the rostral anterior cingulate cortex (rACC) (Petrovic 2002). Placebo increases blood oxygen level–dependent (BOLD) activation in the rACC and periaqueductal gray (PAG) (Wager et al. 2004) and enhances their connectivity (Bingel et al. 2006), while naloxone administration reduces placebo effects on pain, BOLD activation, and rACC-PAG functional connectivity (Eippert et al. 2009a). Given that the endogenous opioid system regulates descending pain modulation (Fields 2004), placebos might regulate pain by blocking ascending input. If so, they should also affect nociceptive processing in the spinal cord. Functional MRI studies do indicate that placebos can modulate spinal activation (Eippert et al. 2009b), but some studies fail to see placebo effects on spinal nociceptive reflexes (Roelofs et al. 2000; for a complete review on spinal placebo effects, see Eippert & Büchel 2013).

## SUMMARY POINTS

1. Learning about pain helps organisms update expectations and avoid harm.
2. Learning and expectations directly shape pain and brain responses in both pain-specific brain circuits and those involved in salience, affect, learning, and cognitive control.
3. Pain is influenced by both verbal instructions and experiential learning.
4. Verbal instructions can shape aversive learning, but some circuits may be impervious to instructions.
5. Subjective expectations should be considered separately from learning and instructions.
6. Instructions can enhance placebo analgesia and responses to active analgesic medications.

## FUTURE ISSUES

1. Is pain relief inherently rewarding? Future studies should continue to examine the relationship between reward and pain, as this link may contribute to the addictive propensities of opioid analgesics.
2. Translational work should investigate potentially pain-specific modulatory mechanisms using preclinical models of aversive learning, pain, and placebo analgesia.
3. How do endogenous opioids and dopamine contribute to pain and its modulation? Are the systems independent, or do they interact?
4. Can pain-predictive cues serve as a model for placebo analgesia and nocebo hyperalgesia, or do they reflect separate mechanisms?
5. What factors determine whether expectations have additive or interactive effects with active analgesics in the balanced placebo design?
6. How does the social relationship between patient and provider impact the effect of instructions and expectations?
7. Future research on descending pain modulation and the role of endogenous opioids should reconcile research indicating that placebo can modulate spinal cord responses (Eippert et al. 2009a) with findings that placebo analgesia does not modulate the neurologic pain signature (Zunhammer et al. 2018).
